# Patient experience of a psychiatric Mother Baby Unit

**DOI:** 10.1371/journal.pone.0198241

**Published:** 2018-05-30

**Authors:** Tanya Wright, Tanisha Jowsey, Josephine Stanton, Hinemoa Elder, Suzanne Stevens, Trecia A. Wouldes

**Affiliations:** 1 Department of Psychological Medicine, Faculty of Medical and Health Sciences, University of Auckland, Auckland, New Zealand; 2 Child and Family Unit, Starship Hospital, Auckland District Health Board, Auckland, New Zealand; 3 Centre for Medical and Health Services Education, Faculty of Medical and Health Sciences, University of Auckland, Auckland, New Zealand; 4 Te Whare Wānanga o Awanuiārangi, Whakatane, New Zealand; Universite de Bretagne Occidentale, FRANCE

## Abstract

**Background:**

Mothers with severe mental illness are vulnerable and engage with services cautiously due to fears of stigma and custody loss. To develop best practice standards and patient-centred services, the subjective experience of those who use it must inform service improvement and policy.

**Method:**

This study utilised exploratory concurrent mixed methods design with primarily qualitative data. Women admitted between April 2015 and December 2016 to a newly developed psychiatric Mother Baby Unit (MBU) in New Zealand were invited to participate in this study. Qualitative data were collected in three ways: (i) semi-structured interviews incorporating Māori concepts of health and wellness by research assistants near discharge; (ii) invitation to provide anonymous feedback in writing using an open format; (iii) unsolicited verbal feedback provided during a home visit three months after discharge. Thematic analysis was undertaken. Demographic and clinical information was collected prospectively for mother-infant pairs during the course of admission and three months post-discharge.

**Results:**

Forty-five people participated in the study. High rates of satisfaction were described. Strengths of the service–as perceived by mothers–included co-admission of mother and infant, staff warmth and availability, transparent practice, inclusion of families, and having a comfortable environment. Mothers described intense distress and confusion, as well as negative self-perceptions when acutely unwell. Infant co-admission and the inclusion of partners and other family members alleviated mothers’ distress. Personal attributes of staff, practical support with caregiving, a range of therapeutic approaches and holistic care were all valued. Feedback collected three months after discharge was the most reflective. Significant inter-ethnic differences were not apparent.

**Conclusions:**

The experience of inpatient care can have lasting influence on recovery and wellbeing. Employing a Māori model of health broadened the holistic nature of enquiry. The approach and timing taken in seeking the views of participants’ yielded different information, all of which is of value to service evaluation and refinement. The findings suggest that keeping mothers and infants together during health service utilisation such as MBUs should be a priority for policy makers and service designers. This approach is consistent with Māori values, combining the importance of whānau relationships (kinship), wairua (spiritual connectivity), hinengaro (the mind) and tinana (physical health). These findings suggest that ‘holistic care’–in this case following a Māori holistic health model–is important in mental health settings.

## Introduction

Perinatal mental illness can have serious adverse outcomes for the women [1,2], their families and their infants [3]. The first few months postnatally carry the highest risk of experiencing a mental illness for the first time, as well as recurrence of pre-existing mental disorders [4]. Mothers experiencing mental illness of a severity requiring inpatient care express a preference for admission to a psychiatric Mother-Baby Unit (MBU), particularly in comparison to general units [5]; and this is widely considered best practice [6,7]. In addition to the usual difficulties and rewards new parenting offers, the experience of mental illness brings further stressors. Shame, guilt and fear that the illness may have an impact on their child, the associated stigma of mental illness, increased rates of custody loss and a lack of services and supports are consistently identified issues [8–10]. Research on MBUs suggests positive outcomes for women [11,12]. However, there is little qualitative research undertaken within MBUs due to the severity of illness in the mothers, which means our understanding of this vulnerable group is limited.

Listening to people’s lived experiences is central to ensuring mental health services support patient choice, agency, and autonomy, and that both they and services are recovery focussed [13]. Patient-orientated research, just like patient-centred medicine, recognises the need to identify the best intervention for each person and that non-medical aspects of health care are important not only to patient satisfaction, but to patient engagement, their understanding and retention in services [14].

International practices and research must be approached cautiously in this field. Patient-centred service delivery is highly contextual and maternity and infant care are deeply culturally imbued [15]. New Zealand is a multicultural country with Māori being the indigenous people. People and services in New Zealand have specific obligations to the people of the land (tangata whenua) which are outlined in the Treaty of Waitangi (Te Tiriti O Waitangi). This founding document is strongly evident in health and social policy. Health disparities have multifaceted historical and social origins [16]. They are further compounded by institutional and internal racism [17]. Services that support wellness, not simply removal of symptoms, are required to meet the Māori holistic view of health and to contribute to addressing health inequity [18]. In determining Māori health, measurement of standard health outcomes alone is insufficient [19].

Whilst Māori are nearly 15% of the population, 24.9% of all births were to Māori in 2015—the year of this study [20]. Overall, 54.2% of births in New Zealand were non-European, with birth rates for Asian women the most rapidly increasing [20]. Durie [21] called for “the development of a type of psychiatry which is firmly grounded in New Zealand identity”. Accordingly, we have undertaken to use a Māori model of health and wellbeing for all participants in order to broaden the holistic nature of enquiry. The Te Whare Tapa Whā model of health incorporates four dimensions: spiritual health (wairua), physical health (tinana), mental health (hinengaro), extended family relationships (whānau) [18]. This model was used to guide initial interviews. It has not previously been employed in a perinatal setting, nor with a multi-ethnic population.

Research undertaken by interview is particularly rare [5], more commonly surveys and questionnaires are employed [22,23]. Qualitative data are needed to provide in-depth understanding [24] that will inform the quality of care. This paper reports the qualitative data component from a mixed-methods study examining maternal psychopathology, infant wellbeing and maternal infant relationships during the course of admission and three months after discharge from a psychiatric MBU. We aimed to explore mothers’ experiences of an MBU in order to provide evidence that will inform policy and improve MBU services; and, in turn, promote patient-centred care that best supports recovery for women who require high level, resource intensive mental health care during their transition to motherhood.

### Context of the study

This study took place in a MBU, with a three-bed facility for mothers and their co-admitted infants, serving an urban population of 1.57 million and an annual birth rate of 21,665 [20]. It is adjacent to a child and adolescent mental health unit, within a paediatric tertiary referral hospital.

Each mother who is admitted to the unit has her own room and a cot or bassinet. There are common shared spaces, including a kitchen, living area, family area, quiet area and play areas for infants. Women all received treatment for their illness which included psychotropic medications, tailored to their symptoms during the course of the admission. This included antidepressants, antipsychotics, mood stabilisers, anxiolytics and sedative medications. One participant received voluntary electroconvulsive therapy. In addition to pharmaceutical treatment, women received psychological interventions as indicated, most commonly cognitive behavioural therapy, mindfulness training and psychodynamic, representational therapies. Mother-infant interventions included video guidance, support in recognition of infant cues and psychoeducation. Hands-on, practical support in the provision of infant care was available from the nursing team. Safe care was ensured, but the primacy of the mother in promoting the relationship and care for her baby was a central philosophical tenet.

The service is family inclusive, allowing partners and family members to stay at night, although there are a limited number of additional beds. Unless determined to be clinically necessary, visiting is not restricted.

## Methods

Following Leech and Onwuegbuzie’s [25] explanation of mixed methods research as multiple types of data concerning the same subject triangulated to reach conclusions, we employed an exploratory concurrent mixed methods design with primarily qualitative data.

### Trial registration

Approval for all aspects of this study was granted by the Health and Disability Ethics Committee (14/NTA/157) on 24/11/2014 and the Auckland District Health Board (6429) on 8/4/2015.

### Recruitment and participants

All English-speaking mother–infant pairs living in the greater Auckland region who were admitted to the MBU for over 4 days between April 2015 and December 2016 were eligible. All women had acute postpartum mental illness, although some had acute exacerbations of existing illnesses, or a deterioration in their capacity to be supported in a community setting.

Written consent to participate in research was sought by research assistants separate from the clinical team after determining that mothers’ mental state would allow interviews to be performed and they were able to competently consent for themselves, and for information pertaining to their infants; and after consideration as to whether participation would confer additional burden at this clinical time point. This was organised at a time which enabled inclusion of partners and family, unless this was not wanted by the mother. In most cases recruitment occurred on the ward. However, if preferred by patients or their families, the research assistants would contact them by phone within a few days of discharge and visit a place of their choice. This was their home in all instances. Recruitment was always undertaken within a few weeks of leaving the unit.

Clinical and demographic information was collected from the maternal interview and review of clinical records and recorded using a tick-box proforma.

The responsible psychiatrists determined the psychiatric diagnosis for mothers as per the *Diagnostic and Statistical Manual of Mental Disorders*, *Fourth Edition*, *Text Revision* (DSM-IV-R) [26]. For the purpose of analysis, diagnoses were grouped into three categories: (i) non-affective psychosis, including schizophrenia, (ii) bipolar disorder and (iii) depression, anxiety and borderline personality disorder (with comorbid depression).

### Research team and reflexivity

Researchers in qualitative research need to recognise their own subjective viewpoints and biases that they bring to the data collection and analysis [27]. The research team for this study included two Pākehā (non-Māori) clinical psychiatrists employed at the MBU, two Pākehā academic developmental psychologists experienced in longitudinal research with mothers and infants following adverse early experiences, a Māori child and adolescent psychiatrist with extensive research experience and a Pākehā medical anthropologist with extensive experience in qualitative and indigenous research, and health service improvement. All members of the research team were mothers.

The principal investigator in this study (the first author) is a psychiatrist at the MBU with whom many participants had been in a therapeutic relationship prior to and during their engagement in the study. This close relationship may facilitate disclosure of internal aspects of experience that researchers unknown to the participants may not have. It is also inescapable that the findings of this research may be influenced by the principal investigator being a clinician with a professional interest in portraying the MBU positively. To mitigate this potential bias data analysis was undertaken by multiple members of the research team including members who were external to the unit and had no identified conflicts of interest. In addition, data analysis and synthesis were undertaken while the principal investigator was on a period of extended leave from the service in order to promote distance and objectivity between the principal investigator and data.

### Data collection

Three approaches to data collection were undertaken to allow a diverse and realist evaluation. When verbal feedback was given, field notes were taken and written verbatim quotes recorded therein.

#### Semi-structured discharge interview

Immediately following the consent process, the research assistants asked about the experience of inpatient care. They asked six questions (Table 1), with four being drawn from the Te Whare Tapa Whā model [28]. Prompting questions were used to elicit further information. This approach was developed in consultation with the Auckland District Health Board Māori Research Committee to broaden the enquiry of health outcomes. Whilst for Māori this describes a holistic view of health, for non-Māori, it recognises that quality of care incorporates non-medical aspects of healthcare and expands the biopsychosocial model.

**Table 1:** Interview questions at discharge from the MBU.

Do you think this admission was helpful for you/them and/or their baby? Has admission been positive for their/ family spiritual health (wairua)? Has admission been positive for their/ family physical health (tinana)? Has admission been positive for their/ family mental health (hinengaro)? Has admission been positive for their/ family relationships (whanāu)? Was there any particular aspect of the admission that you considered most valuable?

#### Written, anonymised data

Research assistants invited anonymous, written feedback by giving participants an addressed and postage prepaid envelope, along with a piece of paper with the following script at the top. ‘Were there any problems or concerns you would like to feed back (anonymously)? Understanding what we do well and what needs to be better is really important to us.’

#### Spontaneous verbal feedback post-discharge

Three months after discharge from the MBU, a home visit was organised by the principal investigator. The initial purpose of this was to collect further quantitative outcome measures. However, participants frequently requested a further opportunity to provide oral feedback on their experience at that visit and this was respected, with the principal investigator writing their verbatim quotes therein. To allow inclusion of these data further ethical approval was sought and obtained.

### Data analysis

Analysis followed broad guidelines for thematic analysis [24,29] conducted in an experiential, realist framework. Two authors (TW and JS) read through the transcribed data looking for recurrent themes and patterns of interest. An initial coding scheme was developed in QSR NVivo Qualitative Software (Version 11), containing concepts, subsidiary concepts and their definitions. Differences were addressed through an iterative review process back and forth between codes and raw data, to reach collective agreement around key and most relevant patterns in participants’ experiences. Eighteen key concepts were developed. Relationships within and between concepts were explored, as were relationships between demographic and clinical information and method of data collection.

Basic descriptive analysis was undertaken using SPSS (Version 24).

## Results

### Sample characteristics

Fifty-two participants were eligible during the study period and 45 consented to participate (86.5%). Participants are described in Table 2. Mother ages ranged from 18–42 years, with a median of 33 years. More than half (28/45) were first-time mothers and most lived with their partner (37/45). A range of ethnicities and primary diagnoses were obtained. In addition to primary diagnoses, there were high rates of Axis 1 and Axis 2 comorbidity (68.9% and 39.5% respectively). Comorbidity was highest in the group clustered together with depression, anxiety and borderline personality disorder (81.5%). Eleven participants (24.4%) were treated under the Mental Health Act during part of their stay.

**Table 2:** Maternal demographic and clinical characteristics.

	N	Mean (SD) or N (%)
**Mother’s age (years)**	45	32.4 (5.93)
**Number of children**		1.58 (0.92)
**First child**		28 (62.2%)
**Living with partner/father of baby**		37 (82.2%)
**Quality of relationship with partner**	37	
**Good, sufficiently supportive**		24 (64.9%)
**Poor, including disclosed violence**		13 (35.1%)
**Ethnicity**		
**NZ European (NZE)**		27 (60%)
**Māori**		9 (20%)
**Pacific Island**		5 (11.1%)
**Asian**		4 (8.9%)
**Indian**		0
**Education**		
**Any tertiary higher education**		24 (53.3%)
**Total household income (NZ quintiles 2015)**		
**Lowest two (<$60,000)**		19 (42.2%)
**Medium ($60,000 - $90,000)**		12 (26.6%)
**Top two (>$90,000)**		14 (31.1%)
**Primary Axis I diagnosis**		
**Schizophrenia/non-affective psychosis**		11 (24.4%)
**Bipolar disorder**		7 (15.6%)
**Depression (with or without psychotic features)**		20 (34.7%)
**Primary anxiety disorder** (including OCD*)		5 (11.1%)
**Borderline personality disorder**		2 (4.4%)
**Length of stay (days)**		23.89 (13.1)

*OCD Obsessive compulsive disorder

The index episode of illness had been present antenatally in 62% of participants. Women were generally admitted early in the postnatal period, with 22.2% of admissions occurring within 4 weeks postnatally, and 48.7% occurring within 8 weeks. Child protection services had received notification of concern for the infant’s wellbeing either before or during admission in 48.9% of cases. The participants are further described in Table 2. We looked for differences in the datasets on the basis of ethnicity, diagnosis and number of children. No significant associations were identified, indicating generalisability of the findings.

The mean length of hospital admission was 23.89 days (± 13.10) and did not vary significantly between different diagnostic groups (F (2, 46) = 1.67, p = 0.203).

At the three-month-home visit, five were not available for follow-up (11.1%). One had moved overseas, two refused ongoing participation. Two mothers had recently had their infants removed from their care by child protection services and they both had experienced significant deterioration of their mental wellbeing. One was admitted to general inpatient care under the Mental Health Act. Whilst the second woman remained at home, her mental health team advised that pursuing a follow-up visit could be detrimental to her. Home visits were undertaken for two mothers who had lost legal custodial rights but continued to be their caregivers. An additional three mothers had voluntarily relinquished the care of their infant to other family members, but were interviewed.

### Overview of feedback from different sources

#### Semi-structured discharge interview

All participants completed the semi-structured interview questions. Overall perceptions were positive, with 43/45 (95.6%) reporting the experience to be positive/helpful for them and for their child. Physical (tinana) and mental health (hinengaro) were the domains considered to have showed the greatest gains and were most comprehensively described by participants. The question pertaining family relationships (whānau) was also generally positive, however, 6/45 participants expressed finding the unit unhelpful in this regard. The domain of spiritual health (wairua) elicited the poorest responses, both with respect to the least improvement as well as 25/45(55.6%) conveying a poor understanding of the question. Results are depicted in Table 3.

**Table 3:** Frequency of response to Te Whare Tapa Whā questions.

	Yes	No	≤10 words	Did not answer question
**Q1 Helpful**	43	2	12	0
**Q2 Spiritual health (wairua)**	19	1	15	25
**Q3 Physical (tinana)**	41	2	11	2
**Q4 Mental health (hinengaro)**	40	1	15	4
**Q5 Family relationships (whānau)**	31	6	10	3

Within this set of data, comments on staff (47 comments) and physical aspects of the unit (size, comfort, noise, food quality) (14 comments) were most frequent, followed by praise for allowing partners to be present on the ward at all times (11 comments), the importance of infant co-admission (10 comments) and valuing support to sleep (10 comments).

#### Written, anonymised data

In addition to the participant interviews, one third of participants (37.7%) also provided anonymised letters by post. Two were explicitly identified as being from a father. In seven of these, feedback was extensive, specific and detailed with recommendations on the physical environment and ward activity program, as well as requests for more psychological therapy. Two responses reported no complaints; however, overall there was considerably more criticism and recommendations in this dataset.

#### Spontaneous verbal feedback post-discharge

Thirteen participants (28.9%) requested an opportunity to provide verbal feedback at the three-month-home visit. This verbal feedback dataset contained considerably richer affective description of their experiences. The desperation and fear they experienced prior to admission, their need for reassurance and sensitive care, as well as the impact illness has had on their lives, world view and relationships were all described.

### Key concepts identified in thematic analysis

We identified seven key themes: 1) staffing and staff interactions, 2) inclusion of mother’s infant by co-admission and therapeutically, 3) involvement of partners and family, 4) physical environment, 5) self-perception across admission and moving towards recovery, 6) aspects raised as therapeutically important to mothers, 7) involvement of child protection services. Additional themes with specific relevance to developing service recommendations were initial difficulties with arrival, the ward at night and the importance of sleep.

#### 1)Staffing and staff interactions

Participants had a lot to say about their perceptions and value of staff, with many of their comments coming at the point of discharge and referring to specific participant-staff incidents (both positive and negative). Clinical staff, particularly nursing staff, featured most often in participant comments, but all ward staff were mentioned, including cleaners, support workers, clerical staff, occupational therapists, psychologists, doctors, psychiatrists and senior management. In many instances contradictory feedback was given. For example, the presence of male nursing staff was valued by some and criticised by others.

Participants especially valued personal attributes of the nursing staff, particularly kindness and caring, as well as their attitudes towards patients.

“*The nursing staff need to be chosen for this ward carefully…it’s kind of like a fish bowl–the nurses need to be respectful/wise discerning people–it can make all the difference to building confidence and getting well*,”

(anonymous feedback)

“*They do not make me feel like a shit parent*,”

*(discharge feedback, NZE, mother with borderline personality disorder and depressive illness)*.

Being able to provide the right amount of caregiving for infants was seen by participants as important. Mothers who did not feel they were able to provide hands-on care themselves wanted practical assistance, particularly to enable them to get more rest and sleep. Others valued being listened to and validated as the parent. Staff presence and availability was described on several occasions as central to their recovery and perceived understaffing or reluctance to help was heavily criticised.

“*Being in the MBU was better than any of the other help I have had, because people asked me what help I needed. I’m not so good at asking for help, so sometimes struggle without it, so this really helped. Especially help getting a rest or helping with (baby) when she was distressed- taking her and dealing with whatever she needed, when I couldn’t understand, or figure out, what she needed*,”

*(discharge feedback, NZE mother with depressive illness)*.

“*[I] really valued having clinical staff available all the time, it really helped me feel safe and that I could trust enough to relax and recover*,”

*(three months post-discharge, NZE mother with non-affective psychosis)*.

“*I felt one staff member really let me down–if I had known I never had to see her again I would have stayed longer*,”

*(discharge feedback, NZE mother with depressive illness)*.

#### 2) Inclusion of infants by co-admission

Being able to be admitted with their infant was raised by about 25% of mothers, particularly first-time mothers. Several explicitly stated it was the only reason they were prepared to be admitted, that separation would have been a barrier to receiving care. The infant was described by participants as positive to their recovery. For some simply staying together was important, for example, *“Having baby on the Unit too is such a big healer*, *just on its own*,” (*discharge feedback*, *NZE mother with depressive illness)*. Others wanted assistance with providing caregiving, particularly with establishing routines and settling and spoke of their growth in confidence being important to their wellbeing. This was more common in first time mothers. Some mothers recognised that their interactions and bonding had been impaired by the experience of mental illness and were able to talk about this concern and address it. Improving the bond and a positive experience of parent-infant therapeutic work was closely related to the theme of recovery.

“*I am much happier now that my son and I have rebuilt our bond*,”

*(discharge feedback, Pacifika mother with non-affective psychosis)*.

“*A whole lot of little things all contributed to my getting better–including regaining confidence with my baby and my ability to manage with her*,”

*(discharge feedback, Pacifika mother with depressive illness)*.

“*Once I began to feel a bit better I was able to learn to see my child’s needs, what my anxiety meant to his experience. It was really hard, but it’s the mother I want to be*,”

*(discharge feedback, NZE mother with depressive illness)*.

#### 3) Involvement of partners and family

Women recognised that their illness affected their whole family and valued their inclusion. Fathers and partners sleeping on the unit, being able to provide care for their infant and recognition of the supportive role fathers played with other children at home was all praised. For some women their support came from other family members, and they appreciated their being welcome on the ward. This time allowed for improved communication with each other, as well as developing better understanding of mental illness and joint planning for the future.

“*Being on the unit has meant I have reunited with my partner and family and know that they are all there for all my children, and me too… when I need them*,”

*(discharge feedback, Māori mother with depressive illness)*.

“*Relationship with partner has come a long way in a positive way; was rocky prior to admission*,”

*(discharge feedback, Pacifika mother with non-affective psychosis)*.

“*I also really appreciated the time we had … for me and my husband. We still aren’t getting on so good, but he has a better idea of what’s going on, and how to help, so we argue less… and we know that is really important for the kids*,”

*(discharge feedback, NZE mother with non-affective psychosis)*.

“*My mother and I now have a plan if I become unwell again*, *to manage my situation*, *now that we know more*,”

*(discharge feedback mother with bipolar disorder)*.

#### 4) The physical environment

Specific suggestions for improving the physical environment were common, notably from father participants. Aspects of the environment that increased participant perceptions of comfort and homeliness were identified by participants as being highly valued. Participants offered specific suggestions for further enhancing this: with night lighting, hooks for clothing, side tables, access to exercise facilities, and double beds. The key environmental complaint was noise, which was primarily a consequence of the site of the unit. They reported that noise adversely impacted on their sleep, which they acknowledged was important to improving their mental state.

#### 5) Self-perception across admission and moving towards recovery

During the visits in mothers’ homes, three months after discharge, participants expressed more thoughts and feelings about their experiences of their mental illness and with treatment within the MBU. The experience of acute perinatal mental illness was distressing and disorientating. They expressed the extent of their desperation and fears prior to admission, both for themselves and for their infants.

“*When I was at home and thinking that I needed to get help I was so afraid. I thought I could go to a dark place and my children would be taken away from me. I thought that was the price I would have to pay for getting help. I knew I had to do it anyway because I had so many bad thoughts I knew I would kill myself*,”

*(three months post-discharge, NZE mother with depressive illness)*.

“*When I got there, I felt very disorientated and confused, and worried about why someone had to be with me, I thought I must be an unfit mother. I didn’t know why I was there*,”

*(three months post-discharge, NZE mother with depressive illness)*.

Women were self-critical, particularly with respect to their incapacity to provide care to their baby. They valued non-judgemental care which was sensitive to this self-perception.

“*I had not bonded at all with my baby and I was ashamed about that. Every time I saw her I felt guilty and so did not want to be near her*,”

*(discharge feedback, NZE mother with depressive illness)*.

“*Shame is a big one, mothers should be able to look after their child, and when you don’t, and can’t, you feel like a monster, sub-human*,”

*(three months post-discharge, NZE mother with depressive illness)*.

Subsequent to leaving hospital and with symptom resolution in particular, women described the profound impact the experience had on their lives, their increased awareness of mental illness in others, feelings of empowerment and altruism.

“*I’m stronger and now I think I have choices. I tell people what happened to me and they often tell me they have had the same thing but never talk about it. (My partner’s) boss was one and he said, “Good on you girl”. This helped (my partner) decide to get help, too. I know people are not always proud of what they have been through, but I am, because I have come through stronger, and now I can use these skills for my friends, husband and baby… so we made a real change in our lives*,”

*(three months post-discharge, Māori mother with depressive illness)*.

#### 6) What was specifically raised as being important therapeutically?

Time, support and staff availability were very frequently raised and praised. Women valued physical health care, particularly being able to go for a walk, getting adequate sleep and having access to a General Practitioner who was available and addressed the physical health needs of both mothers and infants during the course of admission.

Inpatient settings with multidisciplinary teams allow for a range of therapeutic modalities to be available and individually determined. The variability and usefulness of these was clear in the feedback received, as well as their value. Many asked for more non-pharmacological therapeutic input and activities, and only one participant stated that “talking was tiring.” Positive feedback included the importance of learning new skills, specifically mindfulness and Cognitive Behavioural Therapy (CBT).

“*I’m now processing thoughts rather than having them raging through my head. Learnt new ways to cope and handle my thoughts*,”

*(discharge feedback, Pacifika mother with non-affective psychosis)*.

“*Finding time and space to build my skills meant I actually did it–which is what I needed to do*,”

*(three months post-discharge, NZE mother with anxiety disorder)*.

In addition to skills-based approaches, participants were interested in psychodynamic formulations and mother-infant relational therapies; and using this knowledge to improve their parenting.

“*I was able to heal a lot with my past and get clarity about where I was going*,”

*(discharge feedback, Māori mother with depressive illness)*.

“*Being able to be taught how to interact with the baby has helped me. She and I are learning together. I want to learn to not emotionally neglect her and to respond well to her–because I was emotionally neglected as a child. They are working with me to achieve this*,”

*(discharge feedback, NZE mother with borderline personality disorder and depressive illness)*.

“*I learnt a lot about (baby), about being a Mum. It’s helped me want to be referred to infant (mental health services), to do more therapy, so she doesn’t have to do it in the future*,”

*(discharge feedback, NZE mother with depressive illness)*.

Participants reported mixed views on the benefits of pharmacotherapy. Women were conscious of the potential impact it could have on their breastfeeding or when they became pregnant again in the future. Many requested they be given more information and explanation about the potential risks and benefits. There was recognition that pharmacotherapy helped participants. Olanzapine was identified and drew specific criticism. For example, a participant reported that it “made me feel like I must be really sick, if that was needed *(discharge feedback*, *NZE mother with non-affective psychosis)*.

#### 7) Involvement of child protection services

Only three participants provided feedback on this (in seven comments). It was not always done well. Honest and open communication between staff and participants contributed to maintaining a sense of trust between staff and mothers.

“*I think that you did a good job, because even though you had to involve [the child protection agency], I still trusted you and it’s ok*,”

*(discharge feedback, NZE mother with depressive illness)*.

“*Still getting a lot of anxious thoughts about [the child protection agency]. They were notified by MBU about me and (baby) and I didn’t know that was going to happen or why. [It] just suddenly happened. I stopped trusting the people I was working with from that point*,”

*(three months post-discharge, NZE mother with depressive illness)*.

“*I love the MBU, it’s important, but this [the involvement of the child protection agency] was done badly. I still feel ambushed. It made the whole thing [feel] very differently. They should have been more honest about it and talked to me about it. I needed to understand, but I still don’t. I thought the MBU would be safe to explore my relationship, he is (baby’s) father … and we have been through restorative justice, stopped drugs … I just felt tricked, and a bit bullied*,”

*(three months post-discharge, NZE mother with depressive illness)*.

## Discussion

Similar to other studies in MBUs, admission was viewed positively by participants and the presence of their infant highly valued [5]. Personal attributes of staff received the greatest amount of feedback, which is well recognised as being vital to the experience of inpatient care [5,30]. Few comments concerning staff skills or knowledge were made, which may indicate that participants found kind caring attitudes of staff to be more important than skills or knowledge. Instances in which nurses (in particular) were perceived to be short-staffed, unavailable or critical, was hugely influential to mothers’ experiences and influenced their decision to remain in hospital. For some this persisted three months later and remained influential. Many women had made considerable changes in their lives in this time frame, ending abusive relationships and acting upon their improved recognition of their child’s needs. Some spoke philosophically about the need for better societal support of motherhood, a greater awareness of struggles others face and a sense of greater connectedness to their communities as a result. They reported their experiences of MBU was a positive contributor to feeling connected and enriching their life experience. However, for others, detrimental incidents persisted in their thoughts and undermined their self-agency. Recovery literature recognises the influence interactions with mental health services has on facilitating and impeding recovery [31]. This was emphasised in the feedback given at discharge, that positive helping relationships are important in a lasting way. Staff would benefit from greater recognition of the importance of their interactions with mothers.

Staff having enough time to spend with mothers was important. The complexity of the environment and the multiple processes required of staff at the MBU mean that staff spending quality uninterrupted time with mothers at the MBU is not always achievable and participants acknowledged this and advocated for higher staffing for the MBU to enable this to occur. However, Geertz [32] tells us that people benefit from entering a “community of time” with one another, and that it is through spending that time together that relationships and trust are created. We suggest that time at the MBU would have multi-faceted positive long-term influences on mothers; such as in their trust of staff and services and their willingness to seek help in the future.

Participants in this study raised the issue of felt stigma, which Scambler and Hopkins [33] describe as the perception a person has that others will see and treat them differently because of an attribute they display that is stigmatising. Regardless of whether anyone says or does anything to stigmatise the individual, the individual holds fear of the possibility of such stigma arising. In our study mothers noted ‘felt stigma’ associated in relation to their prescribed medication use (particularly olanzapine). They also reportedly experienced felt stigma as ‘being’ “bad mothers.” Recognition of this self-perception, providing support sensitively and recognising the barrier it presents in accessing mental health care is important to acknowledge. This self-stigma is particularly important in a sample in which there is a high rate of involvement of child protection services and in which four mothers relinquished care of their infants to relatives within a short period of discharge.

Many recommendations for service improvement made by participants are possible, such as bolstering preadmission and medication information processes. Several of their recommendations for changes to the physical environment–such as hooks for clothing in the shower room cannot be implemented due to the need to maintain specifications for safety in an acute psychiatric facility. However, lighting at night and decreasing noise warrant consideration for services building new units. In noisy environments where noise cannot be feasibly reduced the use of white noise may be an option that may help some mothers and infants. In an ideal world we would recommend that services locate/build services with sound management built into the structured environment.

The importance of involvement of the wider family, and particularly fathers, is recognised but not well implemented in mental health services [34]. Whilst fathers prioritise the maternal bond to enable admission to a MBU, this can strain their relationship with their child as well as the couple relationship [35]. In this study mothers supported collaborative approaches with their family and natural support systems. This knowledge may help clinicians better navigate information sharing, particularly when this relates to the wellbeing of the infant. The need to include families (whanau) is central to wellbeing for Māori [28] and indeed other cultures [36]. In perinatal and infant mental health, the psychoanalytic tradition similarly recognises the interdependence and the relational self. This is best exemplified in Winnicott’s famous quote “there is no such thing as a baby … if you set out to describe a baby you will find you are describing a baby and someone,” [37].

### Use of the Te Whare Tapa Whā model

Whilst participation in the research achieved near representative rates of ethnicity, we did not detect significant differences in inter-ethnic feedback. It is likely that this is because the use of a Māori concept of health and wellbeing was not sufficient to elicit important cultural concepts.

Examining cultural experience would be enormously valuable. Many cultural concepts are conveyed in nuanced ways, within the language of a culture, and both protocol and process needed. This necessitates research be undertaken by Māori, for Māori (rangahau kaupapa Māori), and that other cultures also undertake this endeavour in the perinatal and infant field.

The four dimensions of Te Whare Tapa Whā appeared to have resonance with many non-Māori. Attending to physical health, through exercise, rest and nutrition, extensive family involvement and respect for intergenerational traditions were widely valued. Wellness is more than removal of symptoms, it is seen as a dynamic interplay of many elements.

The least well answered of the questions was the enquiry about spiritual health, however, only one person said it wasn’t relevant. For Māori, wairua has a different meaning to that of the Western notion of spirituality, however, that was not discernible in responses. This may be a consequence of having no specific interventions or processes, such as singing (waiata), prayer (karakia) or traditional welcome ceremony (powhiri), in routine use in the MBU. Equally, it may have been the first time this aspect of life was raised. The need for rapprochement of religion, spirituality and mental health is recognised but in limited practice [38].

### Child protection

Three of the mothers described the experience of loss of trust and negative impact when MBU staff involved child protection agencies. This is an important issue in the field and highlights the need for consistently skilful, compassionate and honest dialogue. While the results here must be viewed with caution in terms of data saturation and transferability of data, we include them because such experiences were so influential over the participants’ total impressions and experiences and also because other studies have also described fear of losing custodial care as a barrier to accessing mental health care [8]. Whilst multiagency support, including child protection service involvement, is necessary for some mothers who utilise MBUs [39], it can be significantly detriment to maternal wellbeing. A better understanding of the experiences of these mothers, and those who did lose custodial care of their child would be invaluable.

### Implications for health services and policy

Our research shows that women did not want to be separated from their infants in order to address their mental health needs. This fear is a major barrier to receiving mental health care. In the New Zealand prison system, infants up to 24 months are able to remain with their mothers if it is considered to be in the best interests of the child [40]. Mothers with mental illness should be accorded this same right in policy and practice. Co-admission of infants in MBUs, rather than the traditional practice of “boarding” offers many advantages. It recognises an infant’s vulnerability and their rights as patients. In addition to concern for their infants, women are advocating for consideration of their partner’s and their family’s needs, which is currently constrained in existing service provisions. In order for Māori and other non-European people to accept and access preventative and early intervention services for mothers and infants, cultural practices will need to be embedded at all systems and service levels.

### Study limitations and methodological considerations

Using multiple approaches and time points for data collection was intended to optimise participant choice. It reflects differing stages of their experience and supports data triangulation. Rothbauer [41] suggests that when conclusions from different datasets point to the same finding they contribute to the overall validity of the conclusions made.

Study recruitment and interviewing undertaken close to the day of discharge resulted in high participation. However, it may have been misconstrued by some as a requirement given they were still within the hospital. It had been the intention to reduce this power dynamic by employing research assistants who were experienced in recruitment of vulnerable women and were clinical social workers by training. From a clinical perspective, consent and interviewing were undertaken when women were well enough to make an informed decision, however, few were symptom free. It is possible that for many the transition home was an additional source of anxiety and that the process of participation in research added to this further [22,42]. Feedback pertaining to specific incidents with staff dominated the discourse. Responses were often brief, despite gentle use of prompts.

Anonymous, written feedback required additional effort from participants and families. Participation was lower, as has been observed in other studies [22,23]. The phrasing used asked specifically about problems or concerns and this was elicited. This may be an easier format to give negative evaluation. Despite response bias, it is important to have anonymous avenues for feedback and for people to be empowered to express this. Of interest was the spontaneous feedback we received from fathers, who self-identified as such in writing. It may be that this method is particularly suitable for them. It is a weakness of this study that this was not more explicitly sought and should be explored more comprehensively in the future.

The third data set was unanticipated, collected at people’s request, in their homes. Potential for participatory bias is considerable in this dataset also. One might expect that people in many cultures would find it difficult to bring forward overt criticism in the context of a visit by a researcher who had previously been a clinician involved in their care, or of that of a sick relative. However, this was not the case. Often families went to lengths to be together for this and had made preparations for the visit. Many balanced praise with complaint and did so by suggesting that they were hopeful that their experience would benefit others.

This research makes partially visible the largely hidden worlds of mothers with severe mental illness who access MBUs. Undertaking research in this field is not without difficulty, due to the severity of illness, the mothers’ distress and confusion, and the high long-term stakes–physical, emotional and spiritual–for both mothers and infants. Ethical and practical barriers constrain access to undertaking research with women who are experiencing acute, severe mental illness. This contributes to the invisibility of their needs.

Non-participants and women who were not retained in the sample may be the most vulnerable and the least satisfied by the service. However, as a considerable amount of negative feedback was collected we propose that some such people are, at least partially, represented.

Some research bias is noted. MBU staff were aware that research was being conducted in the unit but in order to minimise the potential for the Hawthorn effect staff were blind to the methodology. Although efforts were made to maintain participant anonymity (through the first two anonymous data collection strategies) there is a possibility that participants held beliefs that they were identifiable and that their participation could influence their care. Participants were assured by the researchers that participation or non-participation in this study would have no effect on their care.

By undertaking research in the services we work in, we also introduce bias which seriously impacts upon scientific validity. However, finding ways to support clinician researchers is important. Not only because clinicians are enriched by the practice, but because patient-centeredness, particularly for vulnerable patient groups, requires a commitment by clinicians. It is unlikely that access to this group of women would have been possible, and recruitment as high if not undertaken by a clinician in the service.

The first dataset had a positive bias, particularly the survey question ‘do you think this admission was helpful?’ and the second anonymous dataset had a negative bias with the question ‘were there any problems or concerns you would like to feedback (anonymously)? Understanding what we do well and what needs to be better is really important to us.’ While at face value these questions may seem to elicit superficial responses, we wanted to provide very structured, simple questions for participants. Use of Te Whare Tapa Whā was recommended for this purpose. Data from multiple sources were triangulated in an attempt to mitigate these biases.

A lack of culturally rich feedback is likely to have been influenced by the researchers and staff not being Māori (with few exceptions in the nursing team). It is possible that having limited cultural competence around Māori cultural safety and wellbeing practices also contributed to the findings. We suggest that local training for staff by kaumātua (Māori elders) on such matters could transfer into culturally informed practices of care that are valued by Māori accessing the MBU. In light of New Zealand’s cultural diversity, consideration to other groups is also needed.

### Conclusions

The MBU plays an important part in the spectrum of services needed by women experiencing perinatal mental illness. It was well-regarded by mothers who used the service and they had invaluable feedback and recommendations for service improvement. The importance of staff interactions and availability is a finding that warrants dissemination. Specific recommendations need to be addressed within this service.

The Māori model of health and wellbeing, Te Whare Tapa Whā, was a useful tool for guiding a more holistic conception of health than standard outcomes measurement. However, it was insufficient in eliciting Māori cultural concepts. Further consideration of the needs of Māori (and other non- European cultures) is needed. Whilst inclusion of families is occurring to some extent, and is valued, it is currently restricted to clinicians sharing information with families. MBUs would be ideal services for the introduction of intervention frameworks such as Te Waka Oranga, in which the knowledge systems of family (whānau) and clinical staff are equal participants [43].

## Abbreviations

DSM-IV-R: Diagnostic and Statistical Manual of Mental Disorders, Fourth Edition, Text Revision
MBU: Mother Baby Unit
OCD: Obsessive compulsive disorder
